# Novel Treatments for Age-Related Macular Degeneration: A Review of Clinical Advances in Sustained Drug Delivery Systems

**DOI:** 10.3390/pharmaceutics14071473

**Published:** 2022-07-15

**Authors:** Yolanda Jiménez-Gómez, David Alba-Molina, Mario Blanco-Blanco, Lorena Pérez-Fajardo, Felisa Reyes-Ortega, Laura Ortega-Llamas, Marta Villalba-González, Ignacio Fernández-Choquet de Isla, Francisco Pugliese, Indira Stoikow, Miguel González-Andrades

**Affiliations:** Maimonides Biomedical Research Institute of Cordoba (IMIBIC), Department of Ophthalmology, Reina Sofia University Hospital, University of Cordoba, 14004 Cordoba, Spain; david.alba@imibic.org (D.A.-M.); mario.blanco@imibic.org (M.B.-B.); lorena.perez@imibic.org (L.P.-F.); felisa.reyes@imibic.org (F.R.-O.); laura.ortega@imibic.org (L.O.-L.); marta.villalba7@gmail.com (M.V.-G.); jfchoquet@gmail.com (I.F.-C.d.I.); francisco-pugliese@hotmail.com (F.P.); indirastoikow@gmail.com (I.S.)

**Keywords:** age-related macular degeneration, sustained drug delivery system, clinical trials

## Abstract

In recent years, the number of patients with ocular diseases is increasing as a consequence of population aging. Among them, one of the most common is the age-related macular degeneration (AMD), a condition that leads to vision loss if it is not treated. AMD is a multifactorial disorder with two advanced forms, dry and neovascular AMD. Currently, although there is no approved therapy that significantly impacts dry AMD progression, several pharmacologic therapies exist for neovascular AMD. Notwithstanding, evidence suggests a suboptimal result in a high number of patients receiving these therapeutic options. Consequently, finding effective strategies is not only a still unmet medical need in dry AMD but also in neovascular AMD. This underlines the need for new drug delivery technologies that can improve the pharmacological action and drug concentration at the target sites. In this regard, sustained drug delivery systems are presented as the most promising therapeutic options in AMD patients. This review summarized the pathogenesis and the current treatment options for AMD, focusing on the emerging ocular sustained drug delivery approaches undergoing clinical trials.

## 1. Pathogenesis

Age-related macular degeneration (AMD) is a disease of the retina that can progress to vision impair and even blindness in the elderly [1]. It is a major cause of irreversible blindness in patients over 50 age in the developed world [2]. Currently, about 196 million people have AMD around the world, with an overall incidence of this degenerative ocular disease growing from 4.2% in patients aged 45–49 years to 27.2% in those with 80–85 years [3]. This incidence is expected to rise steadily due to the increase in life expectancy in the population.

A variety of genetic- and environmental-related risk factors are associated to enhanced incidence and progression of AMD. Age is the main non-modifiable risk factor, whereas smoking is the major modifiable risk factor associated with this pathology [4]. The clinical manifestations of this disorder range from discrete drusen deposits and pigmentary changes in early AMD to either geographic atrophy (GA) or neovascularization in advanced AMD forms. It is important to note that both advanced forms, named dry AMD and neovascular/wet AMD, are not mutually exclusive conditions. Dry AMD accounts for 80–90% of cases, whereas neovascular AMD is responsible for 10–20% of AMD cases, the latter explaining the 90% of severe vision loss related to this disease [5,6].

GA presents as a gradual degeneration of retinal pigment epithelium (RPE), photoreceptors, and choriocapillaris that leads to progressive central vision loss [7,8]. Neovascular AMD is characterized by pathological growth and leakage of the vessels from choroid towards the retina or, less frequently, from the retinal circulation [9], causing exudation, hemorrhage, and fibrosis that can damage the retinal layers leading to rapid central vision loss if untreated [10,11,12,13,14,15]. The pathological process in neovascular AMD is mainly led by the vascular endothelial growth factor (VEGF) [16]. 

## 2. Routes and Barriers for Drug Delivery in AMD

AMD is an ocular disease that affects the posterior segment of the eye; it thus becomes a challenge to find an effective treatment for this disease due to anatomical and physiological barriers that must pass the different proposed drugs [17]. To significantly improve drug transport and absorption to the target tissue, a comprehensive study should be performed based on the characteristics of involved barriers and the physicochemical properties of the candidate drugs (e.g., molecular weight, size, surface charge) [18]. Related to barrier characteristics, the posterior segment of the eye includes the sclera, choroid, RPE, and blood–retinal barriers (BRB). The sclera is composed of a random and negatively charged network of collagen fibers and glycoproteins. Drug permeability through the sclera mainly depends on molecular radius and surface charge [19]. The choroid is a network of fenestrated capillaries and comprises the area between the sclera and inner RPE. The main representative barrier for drug delivery within the choroid is Bruch’s membrane. This membrane favors the exchange of positively charged lipophilic molecules such as some nutrients and ions [20]. The RPE is located between choroid and photoreceptors, and allows the pass of hydrophilic and lipophilic molecules through paracellular and transcellular pathways, respectively [21]. BRB is composed of retinal capillary endothelial cells which regulate the transport of molecules across to maintain homeostasis of the retina. As RPE, BRB represents a barrier for large molecules and different studies reported that molecular weight can determine the possibility to pass through tight junctions between the endothelial cells, so macromolecules are restricted to retinal space [22]. Finally, the vitreal barrier is a fluid-like gel, composed of approximately 99% of H_2_O and trace amounts of collagen fibrils, hyaluronic acid, and other ions. Due to its high composition in water, similar viscosity and diffusion coefficients in the vitreous humor are expected, restricting positively charged molecules [23].

**Figure 1:**
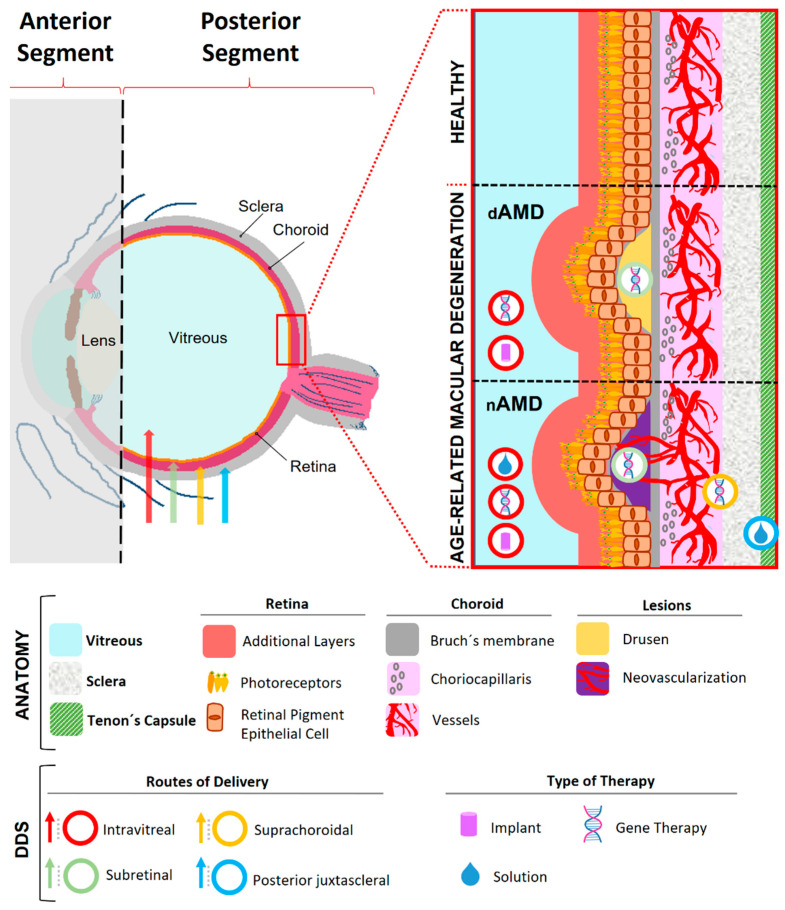
Schematic illustration of eye anatomy and drug delivery systems in Age-Related Macular Degeneration. dAMD: dry Age-Related Macular Degeneration; nAMD: neovascular Age-Related Macular Degeneration; DDS: Drug Delivery Systems.

Within the literature, different studies showed that the intravitreal and periocular routes are the preferred routes for drug delivery [24]. Although the intravitreal route can allow access for delivering drugs next to the target tissue, certain risks are present such as retinal detachment or endophthalmitis [25]. On the other hand, for reaching the target tissue, periocular route is a popular alternative and less invasive than intravitreal route. The periocular route targets the area surrounding the eye and, therefore, this does not present risks related to intraocular pressure (IOP) and retinal detachment. As compared with intravitreal delivery, this method is more effective for delivery to the external area of posterior eye but, in contrast, it is less efficient at reaching the retina [26]. Among the periocular pathways, the posterior juxtascleral, subconjunctival, suprachoroidal, and trans-scleral routes are the mainly used in sustained drug delivery systems to the posterior segment of the eye (Figure 1) [27].

For further information and details about the routes and barriers for drug delivery in the eye, please refer to the review by del Amo et al. [18].

## 3. Current Treatment Options in AMD

To date, there are no approved therapies that significantly impact the progression of GA [7,28,29], although the use of Age-Related Eye Disease Study (AREDS) supplementation has been shown to diminish AMD progression [30,31]. Initial results from AREDS demonstrated that daily supplementation with high antioxidant levels and zinc might decrease the risk of progression to advanced AMD by 25% [30]. Persistent beneficial effects were found after 10 years of follow-up [32]. Nevertheless, AREDS supplementation is not recommended in smoker patients due to beta-carotene increasing the risk of lung cancer [33]. The second AREDS study (AREDS2) was designed to test the addition of lutein/zeaxanthin and omega-3 fatty acids. There was no additional beneficial effect of this new AREDS formulation, although a subgroup analysis indicated that substitution of beta-carotene with lutein/zeaxanthin promoted a slightly decreased risk of progression [31,34]. Notwithstanding, it has been noted that a percentage of patients display an augmented risk of progression while on supplementation, probably due to their genetic background [35,36].

While a treatment for dry AMD remains to be established, different treatment options exist for neovascular AMD such as laser photocoagulation, photodynamic therapy (PTD), and VEGF inhibitors, the latter being the most effective therapy to improve functional and anatomical outcomes in these patients [12,37,38,39,40,41]. Nevertheless, despite the documented benefits of anti-VEGF agents, evidence suggests a suboptimal result in a high number of neovascular AMD patients receiving this treatment [42]. In addition, the burden of repeated intravitreal injections, its associated risks (i.e., endophthalmitis, retinal detachment, and increased IOP, among others), and undertreatment in the real world also contribute to poor visual outcomes in these patients. As a consequence, there is still a high unmet medical need for effective treatment options and strategies in both dry AMD and neovascular AMD.

## 4. Emerging Sustained Drug Delivery Systems in AMD

**Figure 2:**
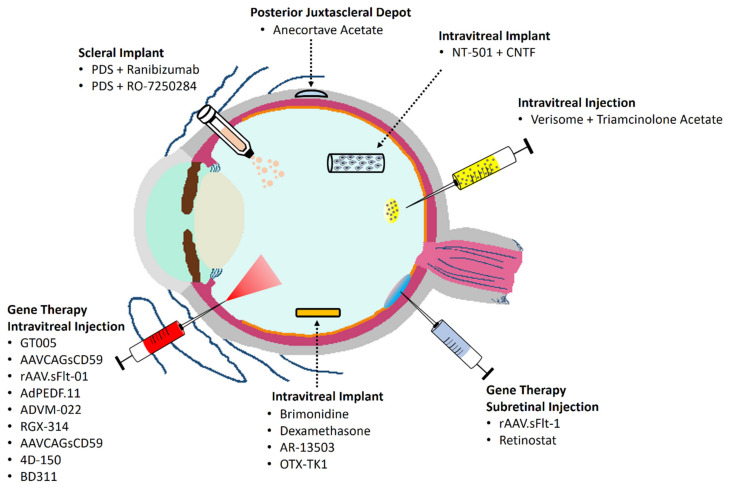
Schematic illustration of emerging sustained drug delivery systems for Age-Related Macular Degeneration in Clinical Trials. CNTF: Ciliary Neurotrophic Factor; PDS: Port Delivery System.

Currently, management of AMD is focused on finding effective treatments and strategies that promote an improved pharmacological action and drug concentration at the target sites. This review offers a comprehensive overview of the most promising therapeutic strategies based on sustained drug delivery systems, specifically on those undergoing clinical trials (Figure 2). Based on the topic, we conducted an initial search using PubMed and Web of Science. Only English publications were included. We incorporated key terms (“drug delivery”, “drug system”, or “drug strategies” plus “macular degeneration”) in our literature search strategy to ensure that all relevant clinical trial manuscripts were found. To minimize the risk of omitting relevant clinical trials, a new search was additionally completed using as key terms the name of each studied drug and macular degeneration. The ClinicalTrials database was also analyzed to find additional information on ongoing clinical trials related to new sustained drug strategies (https://clinicaltrials.gov, accessed on 8 March 2022).

### 4.1. Dry Age-Related Macular Disease

#### 4.1.1. Brimonidine Drug Delivery System

A biodegradable intravitreal implant, the brimonidine drug delivery system (Brimo DDS), has been developed for potential treatment of GA. The implant is administered via intravitreal injection, diffusing brimonidine, an alpha2-adrenergic agonist with cyto/neuroprotective activity, out of the implant into the vitreous humor over a period of several months. A phase IIa clinical trial evaluated the safety and efficacy of Brimo DDS Generation 1 (132 μg and 264 μg brimonidine) in patients with GA from AMD [43]. Participants were randomized in a 2:2:1 ratio to receive treatment (day 1 and month 6) with Brimo DDS 132 μg (*n* = 49) and 264 μg (*n* = 41) or sham procedure as control (*n* = 23) in the study eye and were followed up for 24 months. The primary efficacy endpoint was the change in GA lesion area from baseline at 12 months. Brimo DDS (Generation 1) was well tolerated and proved to reduce GA lesion growth. In fact, both Brimo DDS decreased mean GA lesion area as compared with sham, showing differences between groups at 3 months. In patients with baseline GA lesion area ≥ 6 mm^2^ (66% of patients), lesion area and radius were significantly decreased with Brimo DDS 132 μg and 264 μg respect to sham at 12 months. Related to safety measures, Brimo DDS proved a favorable safety profile, with adverse events usually related to injection procedures. In addition, a large phase IIb study (BEACON) [44] was conducted to evaluate a modified formulation of the implant, Brimo DDS (Generation 2) 400 μg, administered every 3 months from baseline (day 1) through month 21. Sham treatment was used as control. The primary endpoint was the change in GA lesion area from baseline. The BEACON study was stopped due to the rate GA lesion progression being slow (approx. 1.6 mm^2^/year) in the enrolled population, which had a mean baseline lesion area of ~5 mm^2^. Notwithstanding, Brimo DDS significantly reduced the GA progression at 24 months.

#### 4.1.2. NT-501 Implant

Ciliary neurotrophic factor (CNTF) is a member of the interleukin (IL)-6 family of neuropoietic cytokines that has been noted to protect photoreceptors during retinal degeneration in animal models [45,46,47]. Its biological activities are mediated through a heterotrimeric complex formed by CNTF receptor alpha (CNTFRα), glycoprotein 130 (gp130), and leukemia inhibitory factor (LIF) receptor beta, as well as downstream signal transduction pathways [48]. The delivery of CNTF to the retina is a significant challenge due to the BRB. To overcome this challenge, encapsulated cell technology (ECT), particularly the NT-501 implant, was developed to provide a sustained drug delivery system right into the vitreous cavity [49]. ECT consists of genetically engineered cells that express a select therapeutic protein at a regulated delivery rate. These cells are then entrapped within a semipermeable polymer membrane and implanted into the vitreous cavity.

A pilot, proof of concept, phase II trial has already shown promising results for encapsulated cell-based CNTF in GA [50,51]. The 1 mm diameter and 6 mm long devices were loaded with two independent cell lines that released CNTF at low and high doses and were implanted in 39 patients: twenty-seven patients received high-dose implants and 12 patients the low-dose implants. The control group received sham treatment (*n* = 12). The primary endpoint was the change in best-corrected visual acuity (BCVA) at 12 months. No serious implant- or procedure-attributed adverse events were reported over the 12 month period. In addition, no CNTF, anti-CNTF antibodies or anti-encapsulated cell antibodies were detected in serum samples of these patients. CNTF treatment promoted a dose-dependent augment in macular volume. This change was also associated with a visual acuity stabilization from baseline at 12 month follow-up. In a subgroup analysis of patients with baseline BCVA at 20/63 or better, the high-dose NT-501 implant group displayed 0.8 mean letter gain, whereas combined low-dose/sham treatment group showed 9.7 mean letter loss. The devices explanted over the study period presented healthy cells and stable CNTF production.

#### 4.1.3. Ongoing Clinical Trials

A phase I/II clinical trial [52] is currently evaluating the safety, the dose response, and efficacy (anatomical and functional visual results) of three GT005 doses, a recombinant non-replicating adeno-associated viral vector encoding a human complement factor. In addition, a recently completed phase I clinical trial [53] assessed the safety, tolerability and the dose response (low, mid, and high dose) after a single intravitreal injection of AAVCAGsCD59, an adeno-associated viral epitope 2 (AAV2) vector-encoding soluble CD59. This soluble recombinant protein mimics natural CD59 and blocks the membrane attack complex (MAC) formation, thereby protecting retinal cells responsible for central vision.

### 4.2. Neovascular Age-Related Macular Disease

#### 4.2.1. Ranibizumab Port Delivery System

The port delivery system (PDS) is a small, durable, refillable, non-biodegradable drug delivery device implanted into the eye that aims to reduce the burden of repeated intravitreal injections and the undertreatment in neovascular AMD. It is implanted through a small incision in the sclera at pars plana and is designed for continuous extended release of ranibizumab via passive diffusion into the vitreous cavity [54]. A phase I trial was conducted to determine the safety of the refillable drug delivery implant [55]. The PDS filled with 250 μg of ranibizumab was implanted in 20 treatment-naïve neovascular AMD patients. Patients were followed monthly for treatment for a year although were monitored to demonstrate the safety of the implant for a 3 year period. In the last 2 years they received intravitreal injections without refilling the device. The PDS was refilled based on predetermined visual acuity and optical coherence tomography (OCT) retreatment criteria with 500 μg of ranibizumab: 250 μg as intravitreal bolus and 250 μg injected into the PDS. The average number of refills was 4.8 in 12 months. As primary outcome, this study observed 77 adverse events with the most frequent documented effect being conjunctival hyperemia. Three from these adverse events—endophthalmitis, traumatic cataract, and persistent vitreous hemorrhage—were serious and detected in 4/20 neovascular AMD patients. All of them were related to PDS placement. For secondary functional and anatomic outcomes, visual acuity, and anatomical findings, this phase I study observed a visual acuity gain of 10 letters from baseline at 12 months, this improvement being associated with a reduction in mean central retinal thickness. Three of four patients with serious adverse events displayed enhanced visual acuity over baseline at 12 months. In addition, planned device explantation was performed in six patients at month 12. The PDS was noted to be intact and functional, with the implanted PDS being well tolerated in the remaining 14 patients that completed the study.

In the phase II Ladder trial, the safety and efficacy of the PDS with three different ranibizumab formulations (10, 40, and 100 mg/mL) were evaluated and compared to monthly intravitreal ranibizumab 0.5 mg injections in 220 patients with documented response to anti-VEGF agents [54,56]. The study duration was 38 months, with a mean time on study of 22.1 in the combined PDS arms and 21.7 months in the monthly intravitreal ranibizumab injection arm. At the end of the study [56], the PDS was generally well tolerated, with ocular serious adverse events in 9.5% of patients. Vitreous hemorrhage was the most frequent (3.9%) in the overall PDS-treated population, although an 85.7% of them occurred during the postoperative period in the patients implanted before surgical procedure optimization. No serious adverse ocular events occurred in the group treated with intravitreal injections. Related to systemic safety findings, PDS-treated patients showed a profile comparable with monthly intravitreal ranibizumab injection treatment. The median time to first refill was 8.7, 13, and 15.8 months, and 28.9%, 56.0%, and 59.4% patients went ≥12 months without requiring an implant refill in the PDS 10, 40, and 100 mg/mL arms, respectively. Over a mean of 22 months on the clinical trial and consistent with the primary analysis [54], patients in the PDS 100 mg/mL arm had the greatest clinical benefit as compared with the rest PDS treatment arms and presented a visual acuity gain and a retinal anatomy similar to the eyes treated with monthly intravitreal ranibizumab injection, with around 7 times fewer ranibizumab treatments.

Data from the PDS phase III clinical trials provided additional evidence on the safety, tolerability, pharmacokinetics, and efficacy of ranibizumab PDS 100 mg/mL [57,58,59,60]. In the Archway clinical trial [57], a total of 248 patients with recently diagnosed neovascular AMD received treatment with the PDS and 167 with monthly ranibizumab injections. Primary efficacy analysis showed that long-acting PDS-treated patients displayed non-inferior and equivalent visual acuity to current standard therapy with monthly intravitreal anti-VEGF agents. The gain of Early Treatment Diabetic Retinopathy Study (ETDRS) letters was +0.2 and +0.5 from baseline averaged over weeks 36 and 40 in the PDS and intravitreal ranibizumab groups, respectively. Control of retinal thickness was comparable between both groups at week 40. At week 36, mean change in central macular thickness was +5.4 μm in the PDS-treated patients and +2.6 μm in the monthly intravitreal injection treatment. Related to ocular adverse events, 19% in the PDS arm and 6% in the monthly ranibizumab arm were events of special interest and the most of them occurred within 1 month of implantation in the PDS arm. Vitreous hemorrhage was the most frequent (5.2%) in the overall PDS-treated population. Nowadays, the phase IIIb Velodrome clinical trial [59] is assessing the efficacy, safety, and pharmacokinetics of the PDS 100 mg/mL delivered every 36 weeks compared with every 24 weeks, the primary outcome being the change from baseline in visual acuity score averaged over weeks 67 and 72. In addition, the Portal trial [58] is evaluating the long-term tolerability and safety of the 100 mg/mL ranibizumab PDS refilled every 24 weeks or every 36 weeks for approximately 240 weeks in participants who completed either Ladder, Archway, or Velodrome study. Primary outcomes of this clinical trial are the incidence, severity, duration, and causality of adverse events, whereas secondary outcomes include functional and anatomical measurements from baseline up to week 240. Another phase III clinical trial, the Diagrid [60], is now analyzing the effectiveness and safety of the ranibizumab PDS 100mg/mL in a 36-week refill regimen compared with intravitreal aflibercept injections administered per treat-and-extend (progressive extension of treatment intervals up to 12 weeks depending on the clinical findings).

A phase IV study [61], currently recruiting neovascular AMD patients previously treated with anti-VEGF inhibitors other than ranibizumab, is assessing the response to treatment with ranibizumab PDS 100 mg/mL receiving implant refill at fixed 24-week intervals. Additionally, a substudy is conducted to evaluate the impact of the PDS on corneal endothelial cells.

Another neovascular AMD treatment, RO-7250284, has entered phase I trials [62] and is delivered via the PDS and intravitreal injections, demonstrating the opportunity to use this implantable device for other therapies.

#### 4.2.2. Gene Therapy

Recombinant, replicative-deficient AAV vector transduces non-dividing cells, providing long-term protein expression of a transgene product. An attractive therapeutic strategy for prolonged management of neovascular AMD is to transduce retinal cells with rAAV encoding soluble fms-like tyrosine kinase (sFlt-1), a highly potent naturally occurring VEGF inhibitor. A phase I/IIa was carried out to examine the safety and efficacy of rAAV.sFlt-1 [63,64,65,66,67]. In phase I [63,65], patients (*n* = 8) were randomly assigned (3:1) to receive a single subretinal injection of either low- and high-dose rAAV.sFlt-1 (1 × 10^10^ vector genomes (vg) and 1 × 10^11^ vg, respectively), or no gene therapy treatment (control group). All patients were followed up for a year (total of 12 study visits) and permitted retreatment with ranibizumab according to prespecified criteria. The rAAV.sFlt-1 treatment was found to be safe and well tolerated, with vectors being mostly contained within the target tissue and no ocular or systemic adverse effects attributed to rAAV.sFlt-1. As secondary endpoint, this study noted that 67% of patients in the gene therapy groups did not require rescue injections, whereas 33% required only one injection. In addition, rAAV.sFlt-1 groups improved central point thickness and 83% of them gained vision at 1 year. After the month 12 primary endpoint, participants were additionally followed for two more years, with protocol-specified visits at 18 and 36 months [66]. No ocular and systemic safety signals were noted, with stable or improved BCVA in 67% of participants in gene therapy groups. Central point thickness was reduced at 36 months from baseline in all remained patients. Ranibizumab was administered using a treat-and-extend strategy for long-term follow-up period and it was observed that high-dose treatment received 0 injections as compared with a total of 10 and 1 retreatment injections in low-dose treatment and the control group, respectively. In the phase IIa study [64], the safety and tolerability of the highest dose rAAV.sFlt-1 was evaluated in a large, more representative neovascular AMD population. A total of 32 patients were randomly allocated to receive subretinal rAAV.sFlt-1 gene therapy (*n* = 21) or no gene therapy treatment (*n* = 11) for 1 year. Consistent with phase I data, results of the phase IIa study noted no serious gene therapy-related ocular or systemic side effects, ranging from mild to moderate in the gene therapy group. In the gene therapy group, biodistribution of rAAV.sFlt-1 outside target tissue was transient and limit. BCVA was maintained/improved in a 57% of patients after 1 year post-subretinal rAAV.sFlt-1 injection, although no significant between-group differences in BCVA or center point thickness was noted. The median number of ranibizumab retreatments was two for the gene therapy group and four for the control group. In the long-term follow-up period [67], there were no adverse events associated with gene therapy. Limited biologic efficacy signal in terms of BCVA gains and central point thickness reduction was observed, probably due to population size and characteristics of patients.

In another phase I clinical trial [68], intravitreal administration of AAV2 coding soluble VEGF receptor (sFLT01) was shown to be safe and well tolerated over 1 year follow-up. Nevertheless, they observed a variability in expression and anti-permeability activity, possibly due to differences in baseline anti-AAV2 serum antibodies.

The proangiogenic and pro-permeability functions of VEGF can also be countered by other natural proteins generated during the wound-healing process. Some of these proteins are generated by proteolytic cleavage of proteins with other functions, as endostatin, a cleavage product of collagen XVIII, and angiostatin, a cleavage product of plasminogen. Others, including pigment epithelium-derived factor (PEDF), are generated by nonvascular cells involved in wound repair. A Phase I clinical trial tested the safety and expression profile of a lentiviral Equine Infectious Anemia Virus (EIAV) vector-encoding endostatin/angiostatin (Retino-Stat^®^) [69]. Twenty-one patients with advanced neovascular AMD were enrolled and the study eye received a single subretinal injection of 2.4 × 10^4^ (*n* = 3), 2.4 × 10^5^ (*n* = 3), and 8.0 × 10^5^ transduction units (TU; *n* = 15). Participants were followed up for 48 weeks after which they were encouraged to continue a 15 year long-term follow-up study [70]. All three doses were well tolerated with no dose-limiting toxicities. The analysis of immune response and biodistribution showed that Retino-Stat was not outside target tissue. No serious adverse effects were attributed to the vector. Aqueous humor levels of endostatin and angiostatin were augmented in a dose-dependent manner. Participants receiving 2.4 × 10^5^ and 8.0 × 10^5^ TU displayed an enhanced level of endostatin and angiostatin, which peaked at 57–81 ng/mL for endostatin and 15–27 ng/mL for angiostatin by weeks 12–24, and remained stable through the last determination at week 48. Notwithstanding, anatomic and visual outcomes showed that Retino-Stat did not have a therapeutic benefit in the majority of these advanced neovascular AMD patients.

Related to PEDF, a phase I clinical trial tested the safety of an E1-, partial E3-, and E4-deleted adenoviral vector-expressing human PEDF (AdPEDF.11) [71]. Twenty-eight patients were given a single intravitreous injection of AdPEDF.11 with doses ranging from 10^6^ to 10^9.5^ particle units (PU). There were no serious adverse events, and no dose-limiting toxicities. Signs of mild, transient inflammation were noted in 25% of patients, but there was no severe inflammation. IOP was experienced by six participants and was easily controlled by topical medication. At 3 months, the percentage of patients who had no change or improvement in lesion size was 94% in the groups of higher doses (10^8^–10^9.5^ PU) compared with 55% of patients with lower doses (10^6^–10^7.5^ PU). At 6 months, this percentage was 71% in high-dose groups and 50% in low-dose groups. The median augment in lesion size was 0.5 and 1.0 disk areas in the 10^6^–10^7.5^ PU groups, and 0 disk areas in the 10^8^–10^9.5^ PU groups at months 6 and 12, respectively. These results suggest a single intravitreal injection of doses at or above 10^8^ PU of AdPEDF.11 could result in antiangiogenic activity for several months.

Nowadays, new ocular gene therapies are being developed for neovascular AMD. Ongoing gene therapy clinical trials are summarized in Table 1.

#### 4.2.3. Corticosteroid Implants

A. Anecortave acetate

Anecortave acetate is a synthetic angiostatic agent that inhibits blood vessel growth by suppressing the proteases required for vascular endothelial cell migration and does not exhibit glucocorticoid receptor-mediated biological activities [72]. A phase II clinical trial [73,74,75,76] evaluated clinical safety and efficacy of the anecortave acetate for treatment of subfoveal choroidal neovascularization (CNV) secondary to AMD. This trial recruited and treated 128 patients for up to 2 years. All eyes received a posterior juxtascleral depot injection of three different anecortave acetate doses (30 mg, 15 mg, and 3 mg) or placebo (vehicle), with re-administration at 6 month intervals if the masked investigator believed the patient’s lesion could benefit for additional treatment. The primary clinical efficacy endpoint was mean change from baseline in logarithm of the Minimum Angle of Resolution (logMAR) visual acuity at month 12. The data after 1 year of treatment [74] showed that the visual acuity, stabilization of vision, and prevention of severe vision loss were significantly better in patients treated with anecortave acetate 15 mg than placebo. No statistically significant differences were found between low/high dose and placebo in these analyzed parameters. Subgroup analysis of predominantly classic lesions revealed the same results for each of these three measures of visual outcomes. The Independent Safety Committee identified no clinically relevant treatment-related safety issues at 12 months. These data obtained at 12 months of treatment support the conclusion reached at month 6 [73], i.e., that anecortave is both safe and clinically efficacious for the vision outcomes. However, the statistical superiority of anecortave acetate 15 mg over placebo for inhibition of lesion growth at month 6 was not observed at month 12 [73,74]. In addition, the 2 year efficacy results showed that anecortave acetate 15 mg was statistically superior to placebo for vision stabilization and for inhibition of neovascular lesion growth [75]. No clinically relevant serious treatment-related safety issues associated with either the study medication or the procedure for administration were identified by the Independent Safety Committee [75,76].

An additional phase III clinical trial compared 1 year safety and efficacy of anecortave acetate 15 mg suspension to PTD with veteportin in patients with predominantly classic subfoveal CNV secondary to AMD [77]. A total of 530 participants were randomized 1:1 to receive either a periocular posterior juxtascleral depot of anecortave acetate 15 mg every six months combined with a sham PDT treatment every three months if there was clinical evidence of leakage in fluorescein angiography, or a PDT every three months combined with a sham posterior juxtascleral depot procedure every six months as leakage was detected. The primary clinical efficacy endpoint was percent responders at 12 months. Percent responders in anecortave acetate 15 mg and PDT groups were comparable (45% vs. 49%, respectively), with no differences between groups. The month 12 clinical outcomes for anecortave acetate 15 mg were improved in patients that did not have reflux and were treated within the 6 month treatment window, in which the 57% of participants retained vision compared with 38.1% of those with reflux and treatment window longer than 6 months. No serious adverse events associated with the study drug were reported in either treatment group.

B. Triamcinolone acetate

The sustained delivery of triamcinolone acetate, an antiangiogenic and anti-inflammatory agent, can be achieved using Verisome, a liquid drug delivery technology consisting of a variety of excipients (i.e., carbonates, tocopherols, and citrate ester), which combined with the active drug ingredient to result in a controlled release of that drug (IBI-20089). A phase I study was designed to evaluate the safety and evidence of efficacy of the combination of IBI-20089 plus monthly pro re nata (PRN, “as needed”, monthly injections only in case of active disease) ranibizumab in neovascular AMD patients [78]. Patients (*n* = 10) were randomly assigned to receive a single intravitreal injection of IBI-20089 containing triamcinolone acetate (6.9 mg or 13.8 mg) followed a week later by intravitreal injection of 0.5 mg ranibizumab. The primary clinical endpoint was the safety and tolerability of IBI-20089 (6.9 mg or 13.8 mg) when used adjunctively with ranibizumab 0.5 mg at 12 months. No serious related adverse events occurred after 1 year. Ocular adverse events included elevation of IOP in eight patients and progression of cataract in three of them. Related to preliminary evidence of efficacy, all patients decreased central subfield thickness on OCT at 1 month, with a re-treatment rate of 25% after 1 year. Combination therapy resulted in a mean number of three re-treatments at and including month 12. Visual acuity was stable or augmented in 7/10 eyes at 1 year.

C. Dexamethasone

Several phase II clinical trials have been carried out to evaluate the efficacy and/or safety of dexamethasone (DEX) implant 0.7 mg as an adjunctive therapy to ranibizumab in patients with CNV secondary to neovascular AMD [79,80,81,82]. The clinical trial conducted by Allergan [79] randomized and treated a total of 243 patients with DEX implant or sham procedure (1:1), who received two protocol-mandated intravitreal ranibizumab injections. Participants were followed up for 6 months. The primary efficacy endpoint was injection-free intervals to first as-needed ranibizumab injection. As compared with sham procedure, the patients receiving DEX implant showed significantly greater ranibizumab injection-free interval, with less as-needed ranibizumab injections over the course of the study. Moreover, 8.3% of patients in the DEX implant group and 2.5% of them in the sham group did not require rescue ranibizumab, with statistically significant differences between treatment groups. Related to functional and morphological outcomes, visual acuity, and retinal thickness were similar between DEX implant and sham groups. Safety results showed non-serious ocular adverse events in the study eye for 49.6% of patients treated with DEX implant and 41.5% of them in the sham group. Higher incidence of conjunctival hemorrhage and elevated IOP was reported in the DEX implant group as compared with the sham group. Supporting these conclusions, the study leaded by Rezar-Dreindl et al. [80] showed that the total number of ranibizumab reinjections and the time until first ranibizumab retreatment were reduced in the patient groups treated with a combination of intravitreal DEX implant and ranibizumab (*n* = 20) as compared with the group receiving intravitreal ranibizumab monotherapy (*n* = 20) during 12 month follow-up. In both study arms, ranibizumab was administered at baseline and as needed. A second DEX implant was also allowed for retreatment after at least 6 months. Functional variables (visual acuity and retinal sensitivity) were stable and central retinal thickness was decreased over the observational period in both groups. The safety assessment demonstrated that 55% of patients in the ranibizumab monotherapy and 60% of them treated with DEX implant and ranibizumab were phakic at baseline. After 12 months, 9% patients from the intravitreal ranibizumab group and 33% from the DEX implant/ranibizumab group were referred to cataract surgery. In addition, this clinical trial investigated the course of inflammatory and angiogenic cytokines in the aqueous humor of neovascular AMD patients in both study arms and healthy age-matched controls undergoing cataract surgery [81]. At baseline, an altered cytokine profile was found in neovascular AMD patients as compared with healthy controls. In the intravitreal ranibizumab monotherapy, no inflammatory or angiogenic cytokines were found to be altered by treatment over time, whereas the DEX implant in combination with ranibizumab produced a reduction in VEGF, MIG, platelet-derived growth factor (PDGF)-AA, and transforming growth factor (TGF)-β1. Interleukin 6 and PDGF-AA positively correlated with central retinal thickness changes, and IL-10 and lipocalin-2/NGAL levels showed positive correlations with visual acuity changes.

On the other hand, the OARA study randomized a total of 10 patients to receive DEX intravitreal implants in combination with ranibizumab or ranibizumab monotherapy after a 3 month ranibizumab loading period [82]. Participants were followed for 9 months and ranibizumab was administered as needed for 6 months in both study groups. Primary efficacy outcome was gains in visual acuity. From baseline to the study endpoint, visual acuity gains and central macular thickness reductions were similar for the DEX intravitreal implants in combination with ranibizumab or intravitreal ranibizumab monotherapy. The number of ranibizumab injections between both study arms was similar over the course of the study. Related to safety outcomes, 20% of patients (1/5) developed IOP after DEX intravitreal implants.

#### 4.2.4. Ongoing Clinical Trials

A phase I clinical trial [83] is currently evaluating the safety of AR-13503 sustained release intravitreal implant, a bio-eridible polyesteraminde polymer that provides controlled release of the AR-13503, for treatment of neovascular AMD and diabetic macular edema. AR-13503 is a multi-kinase (Rho Kinase (ROCK) and Protein Kinase C (PKC)) inhibitor that restrains angiogenesis, diminishes retinal fibrosis, and preserves the BRB. Furthermore, another phase I clinical trial [84] is assessing the safety, tolerability, and efficacy of OTX-TK1, a biodegradable implant that incorporates a small tyrosine kinase inhibitor, axitinib, with anti-angiogenic potential.

## 5. Conclusions

The eye shows several barriers with unique characteristics and properties that notably limit and block the AMD treatment. Thus, the therapeutic options currently available remain suboptimal. The application of new therapies with potential to solve these obstacles is therefore an unmet medical need. The present review discussed the status of emerging therapeutic approaches focused on sustained drug delivery systems in clinical trials for dry and neovascular AMD. Based on the data that we presented, the biodegradable intravitreal implants with brimonidine and encapsulated cell-based CNTF therapy are potential strategies for dry AMD patients. In neovascular AMD, the ranibizumab port delivery system, gene therapy, and corticoid implants are shown as promising sustained drug delivery systems in this advanced form of the disease. Notwithstanding, new therapies are being used in ongoing clinical trials, guaranteeing new breakthroughs in AMD treatment and management.

## 6. Future Perspectives

Advances and development of new therapies are an ever-evolving field that could provide a therapeutic option in patients with suboptimal results in clinical practice due to the enhanced pharmacological action and drug concentration at the target sites. This goal could be reached by the improvement in new drug formulations, ingenious mechanisms for drug delivery, as well as the secure implementation of drug delivery devices. On the other hand, considerations such as ensuring the cost-effective use of therapies, improving clinical outcomes, patient safety, and reduction of side effects are also desired. Currently, systematic reviews in this area that synthetize the most recent advances in AMD treatment using explicit and reproducible methods to systematically search sustained drug delivery systems are still needed. In addition, new advances in biomaterials and nanotechnology research are also needed to achieve future breakthroughs for AMD. Continued research into new drug delivery strategies and implant devices could significantly revolutionize patient management, achieving an effective therapeutic treatment for AMD.

## Figures and Tables

**Table 1:** Ongoing gene therapy clinical trials for neovascular AMD.

Name	Synthesized Products	Vector	Phase	Route of Delivery	Sponsor	Trial Registration Number
ADVM-22	Aflibercept	AAV.7m8	I	Intravitreal	Adverum Biotechnologies, Inc. (Redwood City, CA, USA).	NCT03748784
			Extension	Intravitreal	Adverum Biotechnologies, Inc.	NCT04645212
RGX-314	Anti-VEGF Fab	AAV8	I/IIa	Subretinal	Regenxbio Inc. (Rockville, MD, USA).	NCT03066258
			II	Subretinal	Regenxbio Inc.	NCT04832724
			IIb/III	Subretinal	Regenxbio Inc.	NCT04704921
			Extension	Subretinal	Regenxbio Inc.	NCT03999801
			II	Suprachoroidal	Regenxbio Inc.	NCT04514653
			Extension	Suprachoroidal	Regenxbio Inc.	NCT05210803
AAVCAGsCD59	CD59s	AAV2	I	Intravitreal	Janseen Research & Development, LLC (Raritan, NJ, USA)	NCT03585556
4D-150	VEGF-C miRNA + aflibercept	AAV	I/II	Intravitreal	4D Molecular Therapeutics (Emeryville, CA, USA)	NCT05197270
BD311	Anti-VEGF-A	IDL	I	Suprachoroidal	Shanghai BDgene Co., Ltd. (Shangai, China).	NCT05099094

AAV, adeno-associated virus; IDLV, integration-deficient lentiviral vector.

## Data Availability

Not applicable.
